# Immunome Research

**DOI:** 10.1186/1745-7580-1-1

**Published:** 2005-09-20

**Authors:** Nikolai Petrovsky

**Affiliations:** 1 Flinders Medical Centre/Flinders University, Bedford Park, Adelaide, 5042, Australia

## Abstract

Immunology research has been transformed in the post-genomics era, with high throughput molecular biology and information technologies taking an increasingly central role. This has led to the development of a new area of science termed "Immunomics", that encompasses genomic, high throughput and bioinformatic approaches to immunology. In recognition of the increasing importance of this field, *Immunome Research *is a new Open Access, online journal, that will publish cutting edge research across the field of Immunomics. *Immunome Research *will publish a wide range of article types including specialty immunology databases, immunology database tools, immunome epitope research, epitope analysis tools, high-throughput technologies (gene sequencing, microarrays, proteomics), white papers, mathematical and theoretical models, and prediction tools. *Immunome Research *is the official journal of the International Immunomics Society (IIMMS).

## Introduction

The explosive growth of molecular biology and information technology has radically transformed immunology research in the post-genomics era. Gone are the days when it was possible to do cutting edge immunology research without the use of molecular tools or ability to access and analyse data from numerous, diverse databases. Cross-fertilisation between these areas has led to a whole new field, called "Immunomics" which is set to transform the way in which immunology research is practiced. Immunomics lies at the intersection of traditional 'wet lab' immunology research, high-throughput technologies and computational immunology. Computational immunology, or immuno-informatics, draws its origins from areas including theoretical immunology, mathematical modelling, artificial intelligence, database management and sequence-alignment tools. The combination of traditional immunology with immuno-informatics, systems biology, high throughput genomics and proteomic molecular technologies is what makes immunomics so powerful.

Given the increasingly central role of immunomics in immunology research, it is timely that the first journal devoted to original research across the field of immunomics is now launched. *Immunome Research *is an Open Access, online journal, that will publish cutting edge research across the field of Immunomics. *Immunome Research*'s Open Access policy changes the way in which articles are published. First, all articles become freely and universally accessible online as soon as they are published, so an author's work can be read by anyone at no cost. Second, the authors hold copyright for their work and grant anyone the right to reproduce and disseminate the article, provided that it is correctly cited and no errors are introduced [1]. Third, a copy of the full text of each Open Access article is permanently archived in an online repository separate from the journal. *Immunome Research*'s articles are archived in PubMed Central [2], the US National Library of Medicine's full-text repository of life science literature, and also in repositories at the University of Potsdam [3] in Germany, at INIST [4] in France and in e-Depot [5], the National Library of the Netherlands' digital archive of all electronic publications.

Open Access has been described as having four broad benefits for science and the general public. First, authors are assured that their work is disseminated to the widest possible audience, given that there are no barriers to accessing their work. This is accentuated by the authors being free to reproduce and distribute their work, for example by placing it on their institution's website. It has been suggested that free online articles are more highly cited because of their easier availability [6]. Second, the information available to researchers will not be limited by their library's budget, and the widespread availability of articles will enhance literature searching [7]. Third, the results of publicly funded research will be accessible to all taxpayers and not just those with access to a library with a subscription. As such, Open Access could help to increase public interest in, and support of, research. Fourth, a country's economy will not influence its scientists' ability to access articles because resource-poor countries (and institutions) will be able to read the same material as wealthier ones (although creating access to the internet is another matter [8]).

Prior to the launch of *Immunome Research *there was no specialty journal covering the new and rapidly expanding domain of immunomics. Previously, researchers were forced to publish their immunomics research either in general immunology or bioinformatics journals. Such journals do not have ready access to expert reviewers with knowledge across all domains of experimental immunology, high throughput technologies and immuno-informatics. Furthermore, researchers interested in the area of immunomics could not easily access or find relevant papers, which were spread across many different journals.

*Immunome Research *is the official journal of the International Immunomics Society (IIMMS). IIMMS has a rapidly growing membership that has expressed the need to have a high quality specialist journal to provide consistent standards to the field of immunomics and to provide a focal point for growth of this area. IIMMS has established a number of working committees, a key role of which will be to develop white papers in their target areas, thereby providing a framework, structure and standards to ensure rapid and consistent growth of these key areas within immunomics.

*Immunome Research *will publish a wide range of article types including specialty immunology databases, immunology database tools, immunome epitope research, epitope analysis tools, high-throughput technologies (gene sequencing, microarrays, proteomics), white papers, mathematical and theoretical models, and prediction tools. Manuscripts in keeping with the journal's scope can be submitted online through the journal website. Each manuscript will be sent to two independent reviewers who will then provide written feedback, and indicate whether or not it is acceptable for publication.

The difficulties in the development and application of computational immunology tools include the high complexity of immunological processes, the intrinsic imprecision of biological measurements, and the biases and misconceptions inherent to the scientific endeavour. Care needs to be taken that the computational tools are adequate and that they properly model the underlying immunological processes. The biases and misconceptions can easily be encoded in computational tools resulting in misinterpretation of the insights provided by computational approaches. One role of computational immunology is to convert immunological into computational problems; solve computational problem using mathematical and computational approaches, and we can then aim to convert these results into biologically meaningful interpretations.

Bioinformatic tools available for database searching and biological sequence analysis have become increasingly sophisticated. These tools allow quick identification of sequences of interest and provide some bibliographic, taxonomic and feature information. Tools for sequence comparison, motif searching, or sequence profiling assist researchers in identifying biologically relevant sequence similarities and features. A newer generation of computational tools enables the modelling of biological interactions and simulation of laboratory experiments. These tools help researchers to identify and focus upon relevant experiments, thus speeding up the discovery process. Biological databases, sequence comparison tools, and more complex modelling and simulation tools are core resources, therefore, for computer-assisted discovery and data analysis. By providing a forum for publication of scientific articles across the broad domain of immunomics, *Immunome Research *aims to become a valuable resource for the whole immunology research community.
